# The role of nutritional support with probiotics in outpatients with symptomatic acute respiratory tract infections: a multicenter, randomized, double-blind, placebo-controlled dietary study

**DOI:** 10.1186/s40795-023-00816-8

**Published:** 2024-01-04

**Authors:** Pavlo O. Kolesnyk, Iryna H. Paliy, Larysa P. Sydorchuk, Zoriana P. Hoda, Nataliya O. Ivanchenko, Oksana S. Lych, Natalia R. Huley, Oksana I. Matsyura, Zoryana L. Slyuzar, Sergiy V. Gerasymov

**Affiliations:** 1 Family Medicine and Outpatient Care Department, Uzhgorod National University, Uzhgorod, Ukraine; 2 Department of Internal and Family Medicine, National Pirogov Memorial Medical University, Vinnytsia, Ukraine; 3 Family Medicine Department, Bukovinian State Medical University, Chernivtsi, Ukraine; 4 Lviv State Center for Disease Control and Prevention of Ministry of Health of Ukraine, Lviv, Ukraine; 5 Lviv Municipal Non-Profit Enterprise Third City Clinical Hospital, Lviv, Ukraine; 6 Danylo Halytsky Lviv National Medical University, Lviv, Ukraine; 7 MedianaStatistics, CRO, Mykhaila Horynia Str. 15-A, Lviv, 79012 Ukraine

**Keywords:** Probiotics, Respiratory tract infection, Antibodies

## Abstract

**Background:**

A number of laboratory data and clinical studies have shown that probiotic bacteria may be beneficial in respiratory viral diseases. We investigated the role of probiotics in coronavirus disease-19 (COVID -19), post-disease symptoms, and humoral immune responses to viral antigens.

**Methods:**

This was a randomized, double-blind, placebo-controlled, prospective, multicenter study. We included symptomatic patients aged 18–65 years without risk of severe disease, and positive antigen/PCR test for SARS-CoV-2. Patients received (*Bifidobacterium (B.) lactis* BI040, *B. longum* BL020, *Lactobacillus (L) rhamnosus* LR110, *L. casei* LC130, *L. acidophilus* LA120, 5 billion CFU total) or placebo 1 capsule a day for 28 days and recorded symptoms. Three months later patients completed Post-COVID-19 Questionnaire (PCQ-19). On days 0–5 and 28–35, blood was sampled for IgG to nucleocapsid protein (NCP) and receptor binding domain (RBD)/spike 1 (S1) protein. The primary outcome measure was a patient global symptom score on day 10 of observation. The difference between groups was assessed using the Mann–Whitney *U* test.

**Results:**

Seventy-three patients were assessed for clinical endpoints and 44 patients were evaluated for antibody production. At day 10, the median global symptom score (interquartile range) was lower in the probiotic group (0.0 (0.0–2.0) vs. 2.0 (1.0–5.0), *P* < 0.05). The probiotic group had a shorter duration of fatigue and anxiety after COVID -19 (*P* < 0.05) and a greater change in IgG concentration on RBD/S1 (225.9 vs. 105.6 binding antibody units/mL, *P* < 0.05).

**Conclusions:**

Use of probiotics alleviates acute and post-disease symptoms, and improves humoral immune response to viral antigens.

**Trial registration:**

Registered at clinicaltrials.gov as NCT04907877, June 1, 2021.

**Supplementary Information:**

The online version contains supplementary material available at 10.1186/s40795-023-00816-8.

## Background

Acute respiratory tract infections (ARTI) place an enormous impact on patients and primary healthcare system due to their extraordinary incidence. In 2019, the world prevalence of ARTI reached 17.2 billion and accounted for 43.8% of all causes of the global disease burden [1]. Respiratory infections are the most common reason for seeking medical attention, with personal recurrence rates ranging from 2 to 6 times per year [2]. Although usually mild and self-limiting, ARTIs significantly affect work productivity and quality of life [3].

Emergence of severe acute respiratory syndrome coronavirus 2 (SARS-CoV-2) causing corona virus disease 2019 (COVID-19) brought a new challenge, being both common and severe, affecting upper and lower airways with considerable constitutional symptoms. As with other respiratory infections, the management of outpatients with mild COVID-19 without risk of progressing to severe disease, remains supportive and include close observation for early recognition of the life-threatening symptoms, reduction the risk of further SARS-CoV-2 transmission, advising on when to seek an in-person evaluation [4]. Absence of the effective specific measures in most COVID-19 cases serves a rationale for exploration of a new complementary approaches, one of which may be the use of probiotics.

Indirect evidence shows that patients with COVID-19 and diarrhea have more severe disease, increased concentration of inflammatory cytokines, markers of tissue damage, suggesting the intestinal cells can serve an additional entry and reservoir for SARS-CoV-2 [5, 6]. As with type II alveolar cells, intestinal and colonic enterocytes express angiotensin I converting enzyme 2 (ACE2), a receptor though which SARS-CoV-2 inoculates the body [7]. Bifidobacteria and lactobacilli adhering to enterocytes can hypothetically interfere with infection process and disease manifestation [8–10]. Interestingly that bacteria may potentially downregulate amide and peptide metabolism in the gut including angiotensin-converting enzyme 2 (ACE2) [11, 12]. It may explain the reason that cell cultures exposed to probiotics yielded lesser amount of transmissible gastroenteritis coronaviruses [13]. Additionally, the beneficial effects of probiotics in respiratory infections can be realized via several non-specific mechanisms discussed within the gut-lung axis paradigm [14], including enhancement of innate antiviral immune defense [15]. A recent systematic review of 23 randomized clinical trials involving a total of 6950 participants with ARTI, demonstrated fewer cases, shorter case duration, and reduced antibiotic prescription rates in patients taking probiotics [16].

The objective of this study was to assess the role of short-term ingestion of probiotics in mild symptomatic COVID-19, post-disease symptoms, and humoral immune response to SARS-CoV-2 in outpatients.

## Methods

This was a multicenter, randomized, double-blind, placebo-controlled, prospective, parallel-group study of the test dietary supplement (TDS) manufactured for the purpose of the study. Approval was obtained from the ethics committees of all participating sites (Uzhgorod National University, Uzhgorod, Ukraine; National Pirogov Memorial Medical University, Vinnytsia, Ukraine; Bukovinian State Medical University, Chernivtsi, Ukraine; Lviv State Center for Disease Control and Prevention of Ministry of Health of Ukraine, Lviv, Ukraine; Lviv Municipal Non-Profit Enterprise Third City Clinical Hospital, Lviv, Ukraine). The study was conducted in accordance with the principles of the Declaration of Helsinki and the privacy rights of patients were respected in compliance with Good Clinical Practices throughout the study. Before enrollment in the study, eligible participants had protocol-specific health insurance and were familiarized with the study procedures. A signed informed consent was obtained from all patients. This study was registered at ClinicalTrials.gov on June 01, 2021 before the first patient signed the informed consent form (identifier: NCT04907877).

Participants were recruited from the general population in primary care and outpatient practices. Brochures and posters about the study were available at multiple SARS-CoV-2 testing booths in the region. Inclusion criteria were 18–65-year-old men or non-pregnant women with symptomatic COVID -19 lasting no longer than 5 days and confirmed by antigen or PCR testing. Patients were excluded if they were at significant risk for a complicated course of COVID -19 due to hypertension, diabetes mellitus, cerebrovascular disease, immunosuppressive conditions, chronic respiratory, cardiovascular, gastrointestinal, or urinary tract disease, malignant tumors, or systemic inflammatory connective tissue disease.

After enrollment, the investigator performed 6 structured telephone calls/telemedicine visits aimed at patient retention, reviewing diaries/questionnaires, treatment, adverse events (AE), TDS storage and intake, reminding patients of nurse visits, and returning patients’ study records.

### Assignments

We randomly assigned participants to receive probiotic or placebo TDS using a 2 × 2 block randomization method with a 1:1 allocation ratio (http://www.randomization.com).

Preparation of probiotic and placebo TDS, blinding of samples and their delivery were performed by Nordic Biotic Sp. z o.o., Warsaw, Poland. One batch of each TDS was produced with a shelf life of 2 years. The TDS was stored at 4–6 °C until it was delivered to the researchers, where it was stored at room temperature.

One capsule of the probiotic TDS contained NORDBIOTIC™ strains of *Bifidobacterium (B.) lactis* BI040 (DSM 33812) 1.25 × 10^9^ colony forming units (CFU), *B. longum* BL020 (DSM 33815) 0.25 × 10^9^ CFU, *Lactobacillus (L). rhamnosus* LR110 (DSM 33794) 2.00 × 10^9^ CFU, *L. casei* LC130 (DSM 33796)1.25 × 10^9^ CFU, *L. acidophilus* LA120 (DSM 33795) 0.25 × 10^9^ CFU, 5 billion CFU total. Excipients were maltodextrin, dicalcium phosphate, hydroxypropyl methylcellulose, titanium dioxide, microcrystalline cellulose, magnesium salts of fatty acids, and silicon dioxide. The placebo TDS contained the same ingredients except bacteria.

Sequentially numbered tubes, were sent to investigators and distributed to the patients so that the type of intervention was concealed at every step of the TDS handling.

Patients started TDS at any time on the day of enrollment in the study and then once daily before breakfast during the 28-day observation period. Compliance was assessed by reviewing patient’s daily records, by structured telephone calls, by counting the number of capsules remaining in the returned tube, and by comparison with the reported number of capsules taken. The patient was considered compliant if he or she had taken at least 93% of the total dose (26 of 28 capsules).

### Assessments

The Participant Questionnaire was designed to collect baseline demographic, medical and epidemiological information. The Respiratory Illness Diary (RID) allowed patient to report and rate symptoms during the first 28 days of observation. The RID consisted of 12 tabulated COVID-19 symptoms: cough, shortness of breath, fatigue, muscle or body aches, headache, loss of taste or smell, sore throat, congestion or runny nose, nausea or vomiting, diarrhea, abdominal pain/discomfort, reduced appetite [17]. Each symptom could be rated as follows: 0—the symptom is not present (“no problem”), 1—the symptom bothers only a little/mildly (“minor problem”), 2—the symptom bothers moderately (“moderate problem”), 3—the symptom is very bothersome (“major problem”).

Based on the WHO`s definition of the Post COVID-19 condition “as the continuation or appearance of new symptoms for at least 3 months after the initial SARS-CoV-2 infection” [18] the Post- COVID -19 Questionnaire (PCQ-19) was kept for 3 months and listed symptoms that might persist or emerge after the acute phase of the disease (attention deficit, diarrhea, red or “burning” eyes, bone or muscle pain, loss of appetite, weakness/fatigue, depression, decreased physical activity, difficulty working, sleep disturbances, and anxiety). To control for confounding presence of a particular symptom before catching COVID-19, the patients were given an opt to state how much the symptom had changed with the disease (“same”, “worse”, “better”). The PCQ-19 was completed simultaneously with the Post-COVID Functional Scale (PCFS) [19].

### SARS-CoV-2 antibodies

Peripheral venous blood was collected on days 0–5 and 28–35 for detection and quantification of SARS-CoV-2 immunoglobulins G (IgG) specific against SARS-CoV-2 nucleocapsid protein (NCP) and receptor binding domain (RBD)/spike 1 (S1) protein. Blood was collected in vacuum blood collection tubes with enhanced coagulation (BD Vacutainer® Serum Tubes, Becton Dickinson, USA), centrifuged at 2000 rpm for 10 min at room temperature, serum separated and stored at -30 °C for 2 weeks prior to testing. Antibodies were measured using laboratory kits for enzyme-linked immunoassay (Anti-SARS-CoV-2 NCP ELISA IgG, Anti-SARS-CoV-2 QuantiVac ELISA IgG) according to the manufacturer’s instructions (Euroimmun Medizinische Labordiagnostika AG, Germany). Results were expressed per milliliter in relative (RU/ml) or binding antibody units (BAU/ml) for NCP and RBD/S1 antigens, respectively. All tests were performed in the central laboratory (Lviv State Center for Disease Control and Prevention) by the assigned testing laboratory technician (OSL).

### Outcome measures

The primary outcome measure was the patient’s global symptom score on the 10th day of observation, a sum of the individual symptoms. Secondary outcomes included clustered constitutional (fatigue, muscle or body aches, headache, nausea or vomiting, decreased appetite), respiratory (cough, shortness of breath, loss of taste or smell, sore throat, congestion or runny nose), and gastrointestinal (diarrhoea, abdominal pain/discomfort) symptoms at day 10; proportion of patients with and duration of gastrointestinal symptoms from day 1 to 10; daily global symptom score for the first 14 days of observation; case severity according to the WHO’s ordinal severity scale [20]; time to resolution of COVID -19, defined as the day when the patient’s global symptom score is 0; proportion of hospitalizations; percentage of AE; percentage and duration of post-COVID-19 symptoms; PCFS score. Tertiary outcome was the concentration of IgG against NCP and RBD/S1 antigens. No changes to study endpoints were made after the study commencement.

### Statistical methods

Sample size was calculated assuming that the probiotic and placebo groups would differ by 4 units on the primary outcome (e.g., 3 versus 7), with a standard deviation of 5, a type I error rate of 0.05, and a power target of 0.9, resulting in 34 patients per treatment group.

Number (%) and median (interquartile range, IQR) were used to describe proportions and continuous variables. The *Z* test and two-tailed nonparametric Mann–Whitney *U* test were used to assess differences between groups. The time to resolution of COVID -19 symptoms was analyzed by the Kaplan–Meier product limit method, and its significance was evaluated by the Gehan’s-Wilcoxon test for time series. To control for confounding factors, ANCOVA analysis was performed with baseline global symptom score and antibody concentration as covariates. The difference between study groups was considered significant at *P* < 0.05. Sample size calculation and statistical tests were performed with Statistica 9 software (StatSoft., Inc., OK).

## Results

**Fig. 1:**
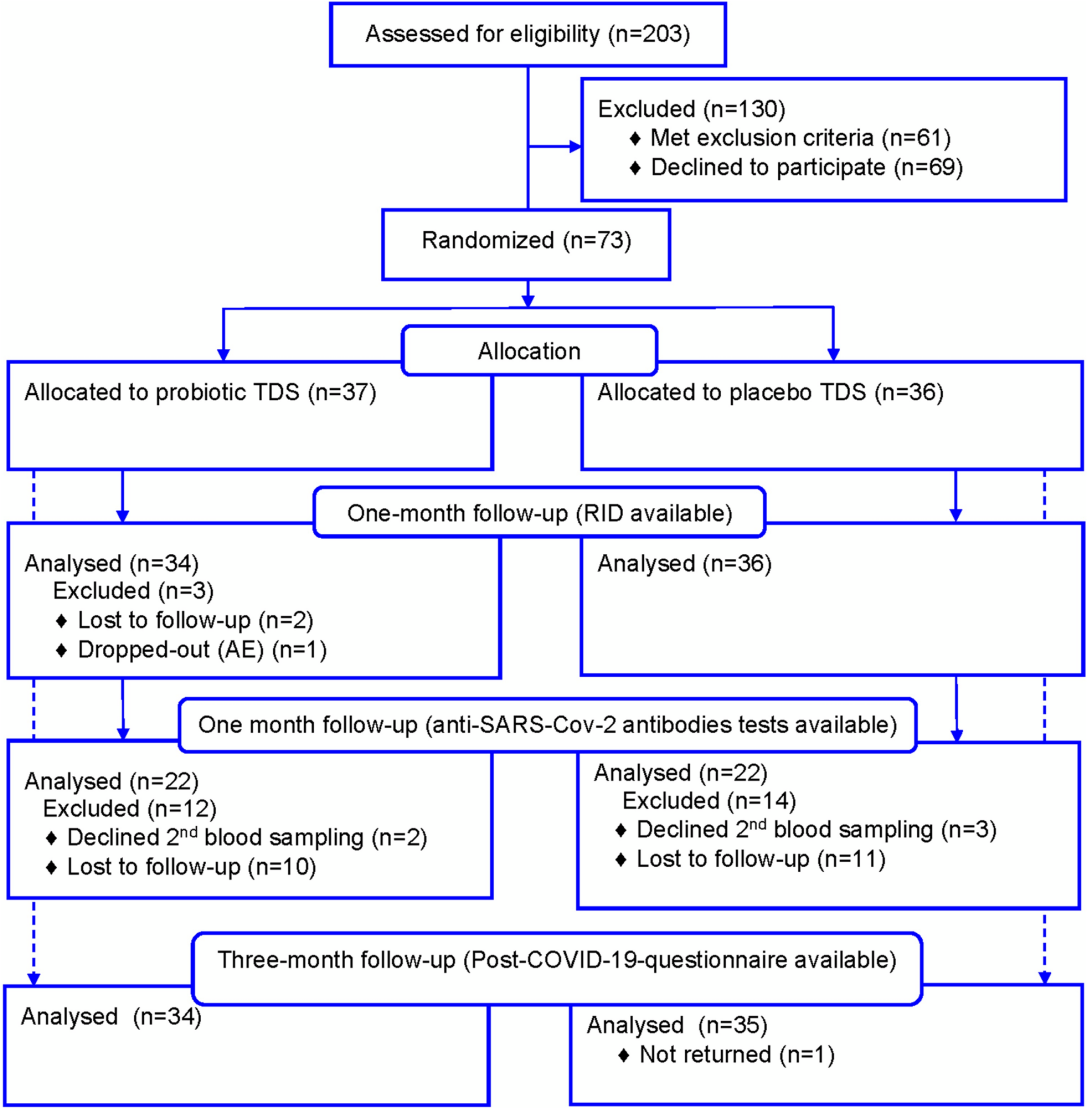
The consolidated standards of reporting trials flow diagram of study participants. TDS, the test dietary supplement; RID, the Respiratory Illness Diary; AE, adverse event. A dash arrow shows participants returned the PCQ-19 in a 3-month follow-up

Screening and enrollment of participants occurred from November 2021 to June 2022 (Fig. 1).

Of 203 age-eligible participants, 30% had other exclusion criteria, and 34% declined to participate. Seventy-three patients were randomized to receive probiotic (*n* = 36) or placebo TDS (*n* = 37). After 28-day observation period, 3 patients in the probiotic group did not return the RID. Five patients refused the second blood draw, and 21 patients were unavailable due to force majeure in the country. One patient in the placebo group did not return PCQ-19.

At baseline, the probiotic group and the placebo group had similar demographic and medical characteristics (Table 1).

**Table 1:** Baseline demographics and clinical characteristics of the study groups

Characteristic	Probiotic group (*n* = 34)	Placebo group (*n* = 36)	*P*—value
Age, years, median (IQR)	44.0 (36.0–48.0)	46.0 (37.5–53.0)	0.264^a^
Weight, kg, median (IQR)	80.5 (69.0–88.0)	78.5 (58.0–94.0)	0.524
Height, cm, median (IQR)	170.5 (165.0–182.0)	169.0 (163.5–177.0)	0.430
BMI, kg/m^2^, median (IQR)	25.9 (23.1–29.8)	26.0 (22.6–28.4)	0.653
Race, Caucasian, n (%)	34 (100%)	36 (100%)	1.000^b^
Gender, male, n (%)	18 (52.9)	19 (52.8)	0.989
Education			
University, n (%)	27 (79.4)	25 (69.4)	0.347
High School, n (%)	6 (17.7)	9 (25.0)	0.459
Incomplete University, n (%)	1 (2.9)	2 (5.6)	0.593
Employment			
Full time, n (%)	28 (82.4)	26 (72.2)	0.320
Part time, n (%)	4 (11.8)	5 (13.9)	0.792
Unemployed, n (%)	2 (5.9)	5 (13.9)	0.272
Welfare			
High, n (%)	6 (17.7)	6 (16.7)	0.914
Middle, n (%)	28 (82.4)	30 (83.3)	0.914
Low, n (%)	0 (0)	0 (0)	1.000
Alcohol consumption^c^, n (%)	10 (29.4)	8 (22.2)	0.496
Smoking status^c^, n (%)	8 (23.5)	5 (13.9)	0.307
Medical profile			
Hypertension, n (%)	4.0 (11.8)	7(19.4)	0.384
Diabetes, n (%)	1.0 (2.9)	2 (5.6)	0.593
Immune deficiency, n (%)	0.0 (0.0)	0 (0.0)	1.000
Respiratory pathology, n (%)	0.0 (0.0)	0 (0.0)	1.000
Cardio-vascular disease, n (%)	3.0 (8.8)	2 (5.6)	0.599
Neoplasm, n (%)	1.0 (2.9)	0 (0.0)	0.308
Rheumatic disease, n (%)	3.0 (8.8)	3 (8.3)	0.942
Cerebrovascular pathology, n (%)	0.0 (0.0)	0 (0.0)	1.000
Hepatic/pancreatic disease, n (%)	0.0 (0.0)	0 (0.0)	1.000
Bowel disease, n (%)	1.0 (2.9)	3 (8.3)	0.338
Renal disease, n (%)	0.0 (0.0)	1 (2.8)	0.335
Epidemiologic history^d^			
Flu-like illness, n (%)	17 (50.0)	15 (41.7)	0.489
COVID-like illness, n (%)	7 (20.6)	5 (13.9)	0.463
Contact with COVID-19 patient, n (%)	24 (70.6)	19 (52.8)	0.136
Symptom scores/proportion			
Respiratory, median (IQR)	3.0 (2.0–5.0)	3.0 (2.0–6.0)	0.810
n (%)	33 (97.1)	35 (97.2)	0.967
Gastrointestinal, median (IQR)	0.0 (0.0–0.0)	0.0 (0.0–0.0)	0.802
n (%)	11 (32.4)	9 (25.0)	0.501
Constitutional, median (IQR)	3.0 (2.0–5.0)	3.5 (2.0–5.0)	0.678
n (%)	33 (97.1)	35 (97.2)	0.967
Global, median (IQR)	7.0 (5.0–10.0)	6.5 (5.0–12.0)	0.948
Antibodies^e^			
Anti-NCP, positive, n (%)	7 (31.8)	9 (40.9)	0.534
Anti-RBD/S1, positive, n (%)	13 (59.1)	14 (63.6)	0.761

*Abbreviations: IQR* Interquartile range, *BMI* Body mass index, *NCP* Nucleocapsid protein, *RBD* Receptor binding domain, *S1* Spike 1 protein

^a^Difference between the groups in the Mann–Whitney *U*-test

^b^Difference between proportions in the Z-test

^c^During the past month

^d^During the past 12 months

^e^Number of blood tests in the probiotic (*n* = 22) and placebo (*n* = 22) groups

A typical patient was a middle-aged Caucasian man or woman with a normal BMI, university degree, full-time employment, moderate income, and comparable alcohol and smoking habits. Hypertension was the most common background disease. Most participants reported having had influenza-like illness and/or contact with COVID-19 patients in the past 12 months, and a substantial proportion had a positive anti-SARS-CoV-2 antibody test result. At baseline, patients were comparable in respiratory, gastrointestinal, constitutional, and global symptom scores. According to patient reports and the number of capsules remaining in the tubes, 5/34 (14.7%) patients from the probiotic group and 10/36 (27.8%) patients from the placebo group missed 1–3 doses of TDS (*P* = 0.186). The number of missed doses was 2.0 (2.0–3.0) and 2.0 (1.0–3.0) in the probiotic group and placebo group, respectively (*P* = 0.594). All evaluable patients took more than 89% of the total dose and were considered compliant with TDS intake.

**Fig. 2:**
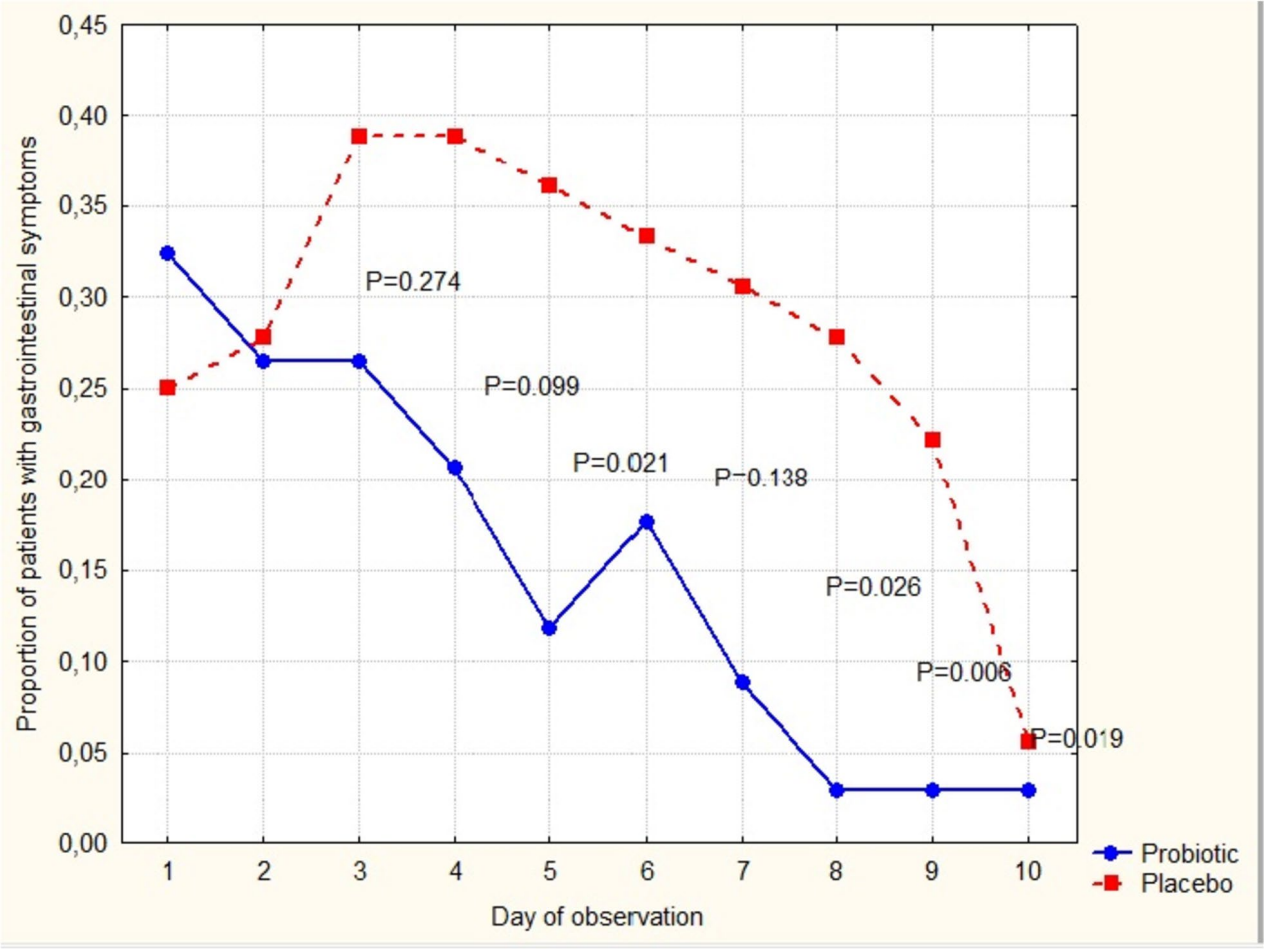
The proportion of gastrointestinal symptoms among patients with COVID-19 during the first 10 days of observation. *P* values are the results of a *Z*-test for difference between proportions at a particular time point

On day 10, the global symptom score was lower in the probiotic group (0.0 (0.0–2.0) vs. 2.0 (1.0–5.0), *P* < 0.05), as were constitutional and respiratory symptoms (0.0 (0.0–1.0) vs. 1.0 (0.0–2.0), *P* = 0.018; 0.0 (0.0–1.0) vs. 1.0 (0.0–2.5), *P* = 0.006), whereas gastrointestinal symptom scores did not differ between groups (0.0 (0.0–0.0) vs. 0.0 (0.0–0.0), *P* = 0.704). However, the proportion of patients with gastrointestinal symptoms was significantly lower in the probiotic group on day 5, 7, 8, and 9 (*P* < 0.05) (Fig. 2).

The duration of gastrointestinal symptoms was not significant between groups (3.5 (1.0–6.0) vs. 5.5 days (4.0–7.0), *P* = 0.102).

**Fig. 3:**
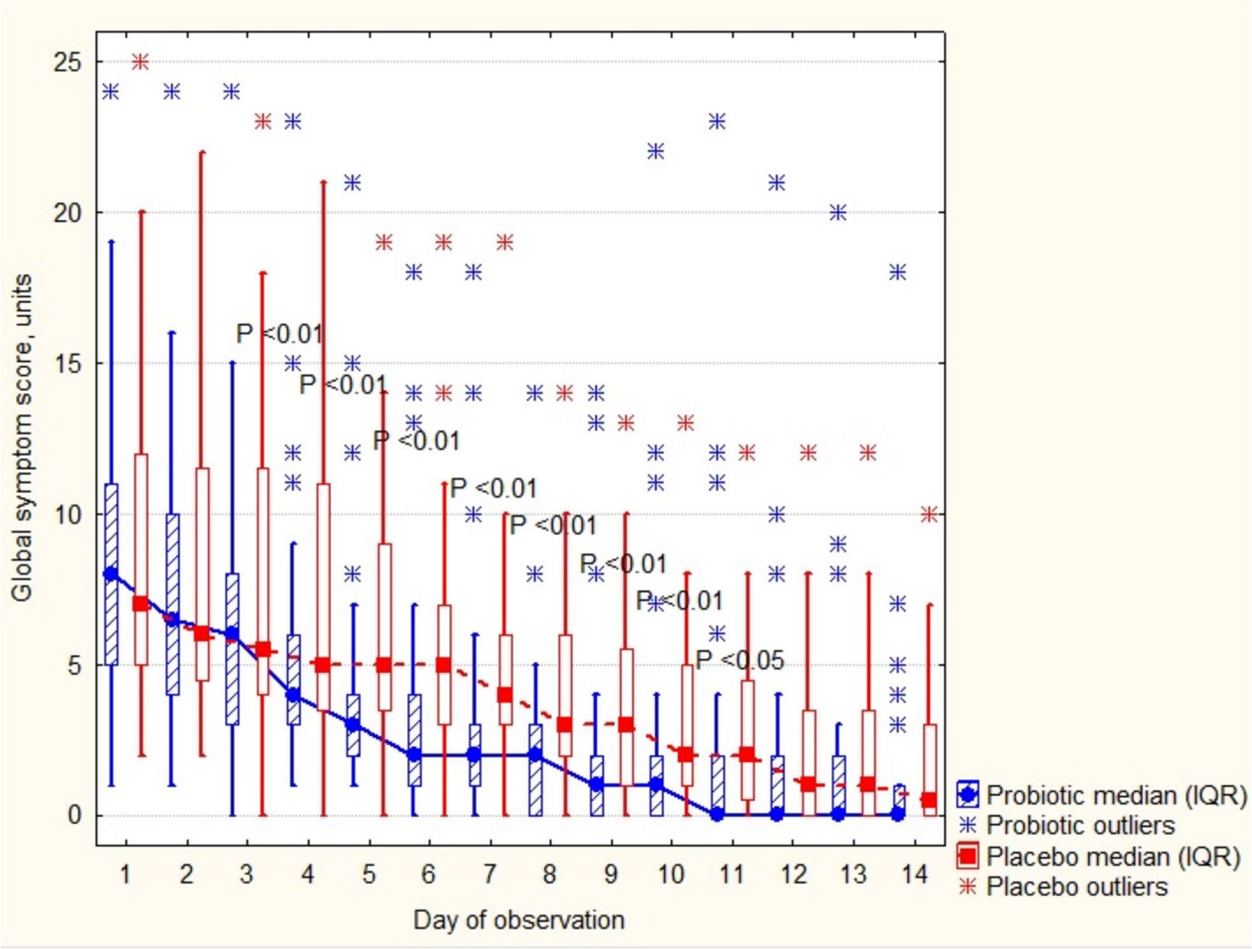
The course of COVID-19 during the first two weeks of observation. P shows difference between groups in the Mann–Whitney *U* test

Daily global symptom scores differed from the 4th to the 11th day of observation (Fig. 3).

According with the WHO’s ordinal COVID-19 severity scale, the proportion of patients with no limitations of activity was greater in the probiotic group (28/34 (82.3%) vs. 20/36 (55.6%) *P* = 0.021). No patients were hospitalized in either group.

**Fig. 4:**
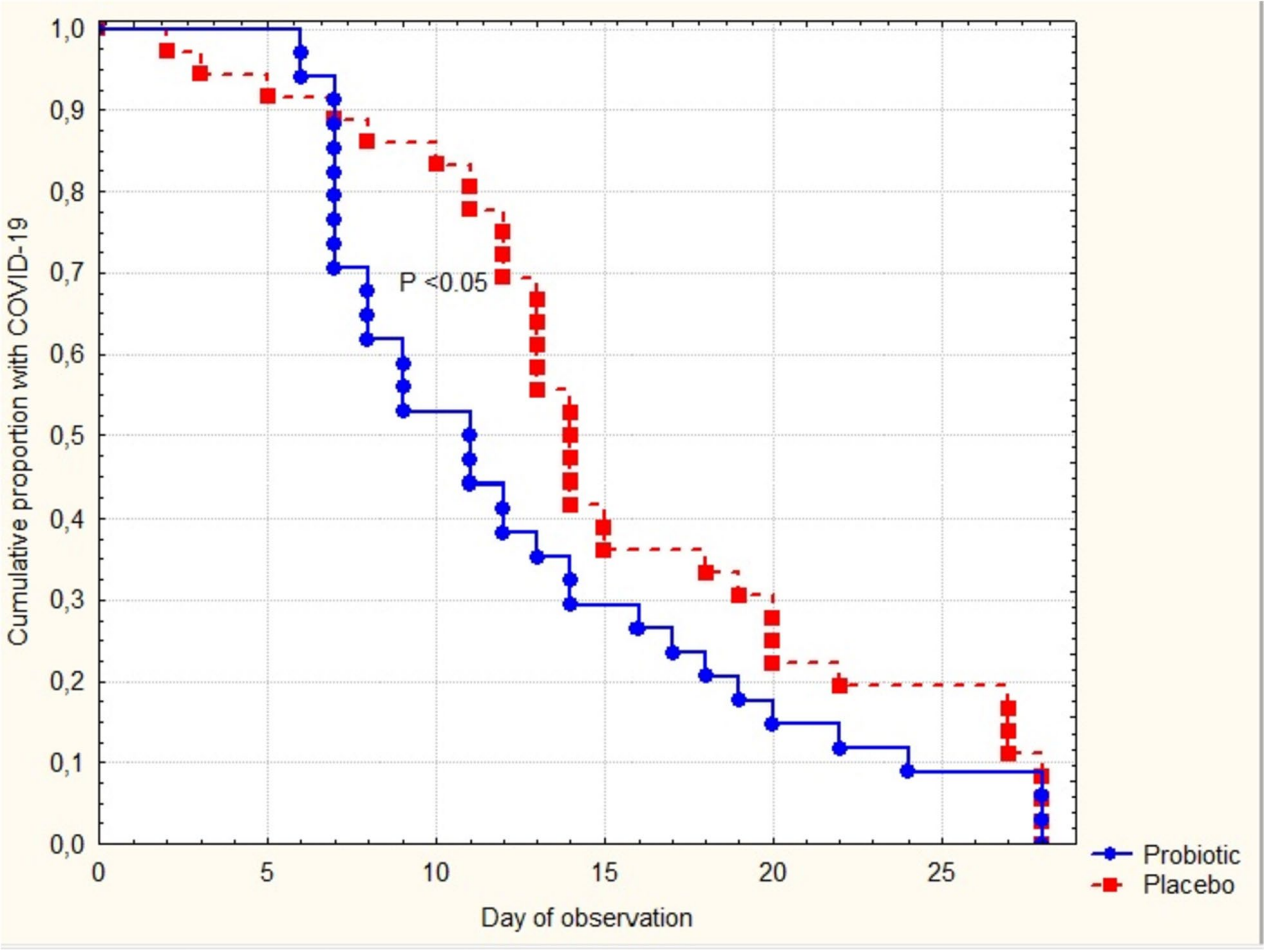
Time to resolution of COVID-19 symptoms. P represents a significance in the Gehan’s-Wilcoxon test for survival curves

The time to resolution of COVID -19 symptoms was significantly shorter in patients taking verum TDS (Fig. 4).

Symptom duration was 3 days shorter in the probiotic as compared with placebo group, 11.0 (7.0–17.0) vs. 14.0 (12.0–20.0) days, respectively (*P* = 0.035).

The percentage and duration of treatment with specific medicines were similar with fewer participants taking antispasmodics and shorter use of throat antiseptics in the probiotic group (Table 2).

**Table 2:** Medication in the probiotic and placebo group during COVID-19

Medication	Probiotic group (*n* = 34)	Placebo group (*n* = 36)	*P*
NSAID, n (%)	7 (20.6)	12 (33.3)	0.239^a^
median days (IQR)	3.0 (1.0–7.0)	4.5 (3.0–6.5)	0.482^b^
Antitussives, n (%)	15 (44.1)	19 (52.8)	0.474
median days (IQR)	5.0 (4.0–7.0)	7.0 (5.0–8.0)	0.241
Nasal decongestants, n (%)	5 (14.7)	3 (8.3)	0.408
median days (IQR)	5.0 (4.0–5.0)	5.0 (4.0–5.0)	0.785
Throat antiseptics, n (%)	7 (20.6)	11 (30.6)	0.347
median days (IQR)	5.0 (4.0–5.0)	6.0 (5.0–8.0)	**0.044**
Antispasmodics, n (%)	0 (0.0)	6 (16.7)	**0.018**
median days (IQR)	-	4.0 (2.0–5.0)	NA^c^
Antidiarrheal, n (%)	1 (2.9)	1 (2.8)	0.968
median days (IQR)	1.0 (-)	1.0 (-)	NA
Antiplatelet, n (%)	1 (2.9)	1 (2.8)	0.968
median days (IQR)	11.0 (-)	4 (-)	NA
Herbs, n (%)	1 (2.9)	2 (5.6)	0.593
median days (IQR)	1.0 (-)	14.0 (6.0–22.0)	NA

*Abbreviations: NSAID* Non-steroidal anti-inflammatory drugs

^a^Difference in proportions in the *Z*-test

^b^Difference between the groups in the Mann–Whitney *U* test

^c^*NA* Not applicable due to absence of variability of the variable

Few AE were reported in the study groups. Two patients in the probiotic group and one patient in the placebo group had developed an urticarial rash that lasted for 1–3 days. Due to rash, one patient in the probiotic group discontinued taking TDS and dropped out, while two others continued TDS with further disappearance of the symptom. Other AEs were abdominal pain with (the probiotic group—1; the placebo group—1) or without diarrhea (the probiotic group—1; the placebo group—1), constipation (the probiotic group—1), and oral enanthema (the probiotic group—1). The initial and worst severities of AEs were grade 1, not affecting activities of daily living. One patient in the placebo group had moderate abdominal pain graded class 2 of severity. All recorded AE were unrelated to the TDS.

The concentration of anti-NCP and anti-RBD/S1 antibodies was not significantly different between groups (Table 3).

**Table 3:** Anti-SARS-CoV-2 antibody (IgG) profile in study participants at baseline (days 0–5) and one-month follow-up (days 28–35)

Antibodies	Baseline			One-month follow-up			Change from baseline		
	Probiotic group (*n* = 22)	Placebo group (*n* = 22)	*P* –*value*	Probiotic group (*n* = 22)	Placebo group (*n* = 22)	*P* -*value*	Probiotic group (*n* = 22)	Placebo group (*n* = 22)	*P* -value
NCP, ratio, units, median (IQR)	0.20 (0.10–1.86)	0.44 (0.28–2.15)	0.080^a^	5.25 (3.12–6.93)	4.13 (3.76–5.20)	0.478 0.178^b^	3.64 (1.64–6.11)	2.97 (1.61–4.10)	0.299
RBD/S1, BAU/ml, median (IQR)	35.2 (3.2–102.4)	62.4 (3.2–198.4)	0.357	312.0 (240.0–384.0)	230.4 (176.0–384.0)	0.072 0.012^b^	225.9 (127.7–317.4)	105.6 (67.8–189.4)	0.012

*Abbreviations: NCP* Nucleocapsid protein, *RBD/S1* Receptor binding domain/spike, *IQR* An interquartile range, *BAU* The binding antibody units

^a^Difference between the groups in the Mann–Whitney *U*-test

^b^*P*-value after controlling the baseline value as a confounding factor in ANCOVA

However, after controlling for baseline values, 28-day probiotic exposure showed a significant increase in RBD/S1 and a tendency towards increase in NCP antibody concentration. Calculation of the change from baseline values also showed an increase in RBD/S1 antibody production in the probiotic group.

Data from the PCQ-19 showed no significant difference between the groups on most variables (Supplementary Table 1).

However, patients in the probiotic group had a shorter duration of fatigue and anxiety and a greater number of patients with lower PCFS scores.

## Discussion

In this multicenter randomized dietary trial of the outpatient adults with COVID-19, we demonstrated that the patients in the probiotic group had lower global, respiratory and constitutional symptom scores on the day 10th. Selection of the 10th day time-point for the outcome measures was made upon the reports that it was critical to the further fate of the patient, improvement or deterioration. The median time from onset of illness to acute respiratory distress was 8–12 days, and admission to the intensive care unit was 9.5–12 days allowing a period of approximately 10 days that the participant theoretically could be observed as an outpatient [21–24]. However, this time point was based on clinical observation of the more aggressive type of SARS-CoV-2 (delta) that was prevalent at the beginning of the pandemics when the study protocol was constructed [25]. According to the local health department updates, the most likely variant of coronavirus circulating in the communities during the study was Omicron B.1.1, with milder manifestation of COVID-19 [26, 27]. Indeed, none of the patients experienced emergencies resulted from respiratory failures (pulse oximetry values varied within normal ranges, data not shown) or in hospitalizations due to other complications. Daily global symptom scores varied within mild disease limits, and progressively decreased for the first 2 weeks with better resolution in the probiotic group.

Along with global symptom score, it`s respiratory and constitutional subsets were lower in the probiotic group on the 10th day of the disease. On the same day, due to their shorter duration, gastrointestinal symptoms were almost absent with no statistical significance between the groups. However, analysis of proportion of patients with gastrointestinal symptoms for the first 10 days was variably lower in the probiotic group, while duration of symptoms only tended to be significant. In the study of hospitalized patients with severe COVID-19 the duration of diarrheal episodes was -2.41 days shorter in patients receiving *B. longum*, *L. bulgaricus* and *S. thermophilus* in a dose of > 6 × 10^7^ CFU a day for 7 days [28]. Along with this finding, the prospective open-label trial evaluating the impact of one-month oral intake of *Kluyveromyces marxianus* B0399 (1 × 10^9^ CFU/day) and *L. rhamnosus* CECT 30579 (1 × 10^8^ CFU/day) showed a benefit in decreasing number of patients without abdominal pain as assessed on the 30th day of observation [29]. The mentioned studies varied in bacterial strains used, type and timing of endpoints for gastrointestinal symptoms, that together with statistically insufficient proportion of patients challenges a direct comparison of the results of our study.

Use of the eight-point WHO’s ordinal severity scale showed greater proportion of patients with no limitation (score 1) and reciprocally fewer numbers with limitation (score 2) of activity in the probiotic group. Reasonably that this finding may be explained by milder disease in the probiotic group according with data retrieved from the Respiratory Illness Diary.

At time of construction of this article, reports on the use of probiotics in clinical trials in COVID -19 patients were sparse. We have located a large randomized clinical study with similar design, prospectively observing clinical course of COVID-19 using a self-reported electronic diary as the data collection form [30]. As with our findings, the authors reported a significant shortening of most COVID-19 symptoms in ambulatory patients taking a mixture *L. plantarum* KABP022, KABP023, KAPB033 and *Pediococcus acidilactici* KABP021 strains in a dose of 2 billion CFU daily for the 30-days starting within first 7 days of the disease. Depending on the particular symptom, use of probiotics was associated with faster improvement in 2.5 through 7.0 days [16]. In our study, the median difference in time to recovery was 3 days for the global symptom score, which falls within the range of the cited report. In the retrospective study of the hospitalized COVID-19 patients, dietary supplementation with *Bifidobacterium*, *Lactobacillus* and *Enterococcus* 1.0 × 10^7^ CFU (strains not specified) each ingredient 3 times daily for 30 days resulted in 3 days faster clinical improvement than in patients without supplementation [31]. Using the mean baseline-follow-up difference of total symptoms per the probiotic and control group on the day 30th of observation, it was shown significant change in proportion of asymptomatic patients favoring the use of probiotics [16].

Other researchers found no probiotic effect in COVID-19 manifestation when evaluating the throat spray containing *L. casei* AMBR2, *L. plantarum* WCFS1, and *L. rhamnosus* GG. The spray was administered in multiple doses, two puffs each, containing approximately 9.5 × 10^8^ CFU, and started within the first 4 days of the confirmed SARS-CoV-2 infection and continued for 14 days [32]. The lack of effect was attributed to the small sample size, the highly variable course of disease in a given patient, and the substantial variability of symptoms among patients. The same study showed a trend toward faster clearance of the virus, with more than three times fewer positive cases in the probiotic group after 3 weeks [19]. Further analysis of microbiome amplicon sequence variants of lactobacilli, indicated significant negative association between the strains and the acute symptom score suggesting that the application of these lactobacilli could result in less acute symptoms in the verum exposure group [19]. An earlier real-life hospital-based observation showed faster recovery from fever, asthenia, headache, myalgia, dyspnea, and an 8-fold lower risk of progression to respiratory failure in patients receiving oral probiotics compared with controls [33]. Intranasal irrigation with *L. lactis* W136 twice daily for 14 days in a small sample of 23 patients aged 18–59 years without concomitant diseases was associated with significantly less fatigue, olfactory dysfunction and dyspnea [34]. The use of *B. longum* ES1, *B. lactis* CBP-001010, and *L. rhamnosus* CNCM I-4036 (> 10^9^ CFU per stick) enriched with zinc, selenium, and vitamin D and administered once daily to hospitalized patients with COVID-19 led to a shorter duration of digestive symptoms and a shorter hospital stay in the cohort of patients with milder pulmonary involvement as evidenced by the chest X-ray [35]. Decrease in calprotectin and C-reactive protein levels, IL-6 in hospitalized patients supplemented with probiotics corroborated their beneficial role in COVID-19 [36, 37]. However, the results of studies exploring upper airway probiotic application or the results of hospital-based studies may not be directly compared with our ones due to the different outcomes measures (recovery vs. improvement, active inflammation markers), severity of the disease (outpatients vs. inpatients), formulations (unprotected probiotics in liquid vs. powder in intestine-soluble capsules), routes of administration (local vs. oral), and areas of action sites (nose/throat vs. small/large intestines).

There is growing number of cell culture and animal studies supporting clinical effects of probiotics in respiratory tract infections, which evolved into a separate concept of the gut-lung axis [14]. Within the concept, gut commensals can distantly signal lungs and influence respiratory system through circulatory transportation of soluble microbial components (peptidoglycans, PG; lipopolysaccharide, LPS) and/or metabolites (short-chain fatty acids, SCFA). In mice model of respiratory syncytial virus infection, PG1505 purified from lactobacteria enhanced innate respiratory antiviral immune response and increased antiviral resistance via activation of Toll-like receptor-3 (TLR-3) [15]. Rectal introduction of bacterial LPS and PG, the TLR agonists, rapidly restored both antibody and T-cell responses to influenza infection in lungs of antibiotically treated mice, further supporting the idea that colonic bacterial products can distantly prime the lungs [38]. Gut commensals synthesizing butyrate (SCFA) from dietary fibers may have an additional potential for sharpening of immunity in respiratory infection. Feces of influenza-infected mice on the high fiber diet, contained almost 140-fold greater concentration of butyrate than controls, which was accompanied with accumulation of alternatively activated macrophages in the lungs, enhanced hematopoiesis of pulmonary anti-inflammatory macrophage precursors (Ly6c– monocytes) in the bone marrow, decreased pulmonary concentration of myeloperoxidase, improvement of symptomatic and survival rates [39]. Furthermore, in mice experimental models lactobacilli elicited systemic effect such as antigen presenting cell migration, enhanced TNF-α and interferon production in airways in response to influenza infection [22, 40–42] Clinical trials evaluating the role of probiotics in respiratory viral infections have demonstrated a probiotic-dependent increase in serum concentration of interferon-γ, NK cell activity, Th1 cell activation, and an increased number of T-helper and T-killer cells [43–45].

In our study, we found that the probiotics increased serum concentrations of anti-RBD/S1 IgG by more than twofold when compared to the placebo. It may account for a faster clinical resolution of COVID-19 symptoms as these antibodies possess neutralizing activity and prevent re-entry of SARS-CoV-2 into host cells upon receptor binding and membrane fusion [46]. Change in median concentration of antibodies against NCP, an important protein which participate in RNA package and virus particle release, showed only a trend toward difference between the study groups probably due to small number of cases tested (22 vs. 22 subjects) and possibly difference of molecular weights of RBD/S1 and NCP antigens. It is academically accepted that larger macromolecules represent better immunogens, as they are more easily processed by macrophages for presentation to lymphocytes, and therefore, can evoke more potent immune responses [47]. Sum of molecular weight of RBD [48] and Spike antigens [49] is higher than that in NCP [50] making theoretically stronger humoral response to RBD/S1complex. Under additional stimulation of the adaptive immune system by probiotics [51], the difference in production of antibodies to antigens with different molecular weights can be even more noticeable and can persist up to the 7th month after onset of the disease[52], suggesting that clinical benefits of probiotics can extend beyond the acute COVID-19 period and influence post-COVID-19 symptoms.

Probiotics decreased duration of post-COVID-19 fatigue and anxiety but did not change their incidence. In the probiotic group, the reduction in fatigability can be linked to the reduction in anxiety, a condition leaving the mind in a constant state of internal tension, mental and physical exhaustion. The importance of anxiety in the post-COVID-19 syndrome is difficult to overestimate due to its high prevalence and even its increasing proportion after acute phase of the disease [53]. After analyzing PCFS data, we also found that probiotics improved patients’ general functional ability. Among others, the PCFS scale contains an “anxiety” variable, that probably played a role in reducing the PCFS score. A meta-analysis of clinical trials on the anxiolytic effects of probiotics revealed a minor but substantial improvement when compared to controlled therapies [54]. Mechanisms behind the effect may be explained within the microbiota-gut-brain axis concept, where probiotics can interact with brain via modification of hypothalamic–pituitary–adrenal pathways [55], synthesis of neurotransmitters (aminobutyric acid, serotonin, dopamine, noradrenaline, melatonin, histamine and acetylcholine) [56, 57], interaction with the nervus vagus [58]. However, the exact anxiolytic role of probiotics in our study remains a field for further exploration.

The strength of our study was its multicenter, randomized, double-blind, placebo-controlled, prospective, parallel group design. Regular phone calls allowed a good patient retention, on-going discussion of the patient self-reported records and thereby obtaining better quality of the source data. The assessment of antibody production revealed one of the potential pathways of probiotic therapeutic effects. However, the failure to evaluate fecal specimens for microbial ecology precluded analysis of the role of the microbiome in the observed effects of probiotics. Neither at baseline nor during the study, the participant had controlled diets that might influence the gut microbiome and influence the outcomes. However, all patients were advised to avoid foods with labelled content of pre- or probiotics, major potential confounders. Another limitation included the use of an invalidated RID and the PCQ-19. However, the endpoints in the mentioned data collection forms were simple, directly related to the disease, and a wording easily comprehended by study participants.

In summary, the results of our study indicate that a short-term use of probiotics plays a role in attenuation of symptoms caused by the SARS-CoV-2 infection, stimulate virus-neutralizing humoral responses, reduce duration of post-COVID-19 anxiety and fatigue. In future studies, we plan to assess a role of the present probiotic strains in prevention of viral respiratory disease as well as their adjuvant properties in anti-viral vaccines.

### Supplementary Information

**Additional file 1: Supplemental Table 1.** The Post-COVID-19 Questionnaire and Post-COVID-19 Functional Scale data after a 3-month follow-up.

## Data Availability

The datasets used and/or analysed during the current study are available from the corresponding author on reasonable request.
