# Supplementary material for: The role of nutritional support with probiotics in outpatients with symptomatic acute respiratory tract infections: a multicenter, randomized, double-blind, placebo-controlled dietary study
Source: BMC Nutr. 2024 Jan 4;10:4. doi: 10.1186/s40795-023-00816-8 (PMC10768308; doi:10.1186/s40795-023-00816-8)
Supplement: Supplementary file 1 — Additional file 1: Supplemental Table 1. The Post-COVID-19 Questionnaire and Post-COVID-19 Functional Scale data after a 3-month follow-up. [file 40795_2023_816_MOESM1_ESM.docx]

Supplemental Table 1. The Post-COVID-19 Questionnaire and Post-COVID-19 Functional Scale data after a 3-month follow-up

| *Item* | *Probiotic group*  *(n=34)* | *Placebo group*  *(n=35)* | P - *value* |
| --- | --- | --- | --- |
| Attention deficit  Present before enrollment, n (%)  Change during COVID-19  Same, n (%)  Worse, n (%)  Present as a Post-COVID-19, n (%)^с^  Duration as a Post-COVID-19, days, median (IQR)^с^ | 2 (5.9)  1 (2.9)  1 (2.9)  7 (20.6)  14.0 (7.0-23.0) | 2 (5.7)  1 (2.9)  1 (2.9)  5 (14.3)  45.0 (21.0-86.0) | 0.976^a^  0.984  0.984  0.495  0.149^b^ |
| Diarrhea  Present before enrollment, n (%)  Change during COVID-19  Same, n (%)  Worse, n (%)  Present as a Post-COVID-19, n (%)  Duration as a Post-COVID-19, days, median (IQR) | 0 (0.0)  0 (0.0)  0 (0.0)  2 (5.9)  16.0 (2.0-30.0) | 1 (2.9)  1 (2.9)  0 (0.0)  2 (5.7)  18.5 (7.0-30.0) | 0.328  0.984  1.000  0.976  0.667 |
| Burning eyes  Present before enrollment, n (%)  Change during COVID-19  Same, n (%)  Worse, n (%)  Present as a Post-COVID-19, n (%)  Duration as a Post-COVID-19, days, median (IQR) | 4 (11.8)  3 (8.8)  1 (2.9)  4 (11.8)  17.0 (7.5-28.0) | 8 (22.9)  3 (8.6)  5 (14.3)  5 (14.3)  32.0 (20.0-32.0) | 0.233  0.971  0.104  0.758  0.190 |
| Bone/Muscle pain  Present before enrollment, n (%)  Change during COVID-19  Same, n (%)  Worse, n (%)  Present as a Post-COVID-19, n (%)  Duration as a Post-COVID-19, days, median (IQR) | 3 (8.8)  1 (2.9)  2 (5.9)  6 (18.2)  28.0 (14.0-60.0) | 2 (5.7)  1 (2.9)  1 (2.9)  6 (17.1)  36.0 (28.0-42.0) | 0.622  0.984  0.542  0.911  0.589 |
| Reduced appetite  Present before enrollment, n (%)  Change during COVID-19  Same, n (%)  Worse, n (%)  Present as a Post-COVID-19, n (%)  Duration as a Post-COVID-19, days, median (IQR) | 3 (8.8)  1 (2.9)  2 (5.9)  5 (14.7)  14.0 (7.0-30.0) | 2 (5.7)  1 (2.9)  1 (2.9)  8 (22.9)  34.0 (17.5-60.0) | 0.622  0.984  0.542  0.393  0.093 |
| Fatigue  Present before enrollment, n (%)  Change during COVID-19  Same, n (%)  Worse, n (%)  Present as a Post-COVID-19, n (%)  Duration as a Post-COVID-19, days, median (IQR) | 4 (11.8)  1 (2.9)  3 (8.8)  13 (38.2)  28.0 (14.0-30.0) | 5 (14.3)  0 (0.0)  5 (14.3)  10 (28.6)  30.0 (30.0-60.0) | 0.758  0.314  0.484  0.401  **0.049** |
| Depression  Present before enrollment, n (%)  Change during COVID-19  Same, n (%)  Worse, n (%)  Present as a Post-COVID-19, n (%)  Duration as a Post-COVID-19, days, median (IQR) | 1 (2.9)  0 (0.0)  1 (2.9)  2 (5.9)  45.0 (30.0-60.0) | 2 (5.7)  0 (0.0)  2 (5.7)  2 (5.7)  36.0 (35.0-37.0) | 0.576  1.000  0.576  0.976  1.000 |
| Decreased physical activity  Present before enrollment, n (%)  Change during COVID-19  Same, n (%)  Worse, n (%)  Present as a Post-COVID-19, n (%)  Duration as a Post-COVID-19, days, median (IQR) | 0 (0.0)  0 (0.0)  0 (0.0)  2 (5.9)  22.0 (14.0-30.0) | 3 (8.6)  2 (5.7)  1 (2.9)  3 (8.6)  60.0 (35.0-90.0) | 0.090  0.167  0.328  0.669  0.200 |
| Decreased work productivity  Present before enrollment, n (%)  Change during COVID-19  Same, n (%)  Worse, n (%)  Present as a Post-COVID-19, n (%)  Duration as a Post-COVID-19, days, median (IQR) | 1 (2.9)  0 (0.0)  1 (2.9)  2 (5.9)  37.0 (14.0-60.0) | 1 (2.9)  1 (2.9)  0 (0.0)  2 (5.7)  22.0 (14.0-30.0) | 0.984  0.328  0.314  0.976  0.667 |
| Sleep disturbance  Present before enrollment, n (%)  Change during COVID-19  Same, n (%)  Worse, n (%)  Present as a Post-COVID-19, n (%)  Duration as a Post-COVID-19, days, median (IQR) | 4 (11.8)  1 (2.9)  3 (8.8)  11 (32.4)  14.0 (7.0-30.0) | 7 (20.0)  5 (14.3)  2 (5.7)  11 (31.4)  30.0 (21.0-51.0) | 0.357  0.104  0.622  0.935  0.193 |
| Anxiety  Present before enrollment, n (%)  Change during COVID-19  Same, n (%)  Worse, n (%)  Present as a Post-COVID-19, n (%)  Duration as a Post-COVID-19, days, median (IQR) | 5 (14.7)  3 (8.8)  2 (5.9)  8 (23.5)  10.5 (7.0-22.0) | 6 (17.1)  3 (8.5)  3 (8.5)  10 (30.3)  45.0 (42.0-56.0) | 0.784  0.971  0.669  0.536  **0.043** |
| PCFS, score  0, n (%)  1, n (%)  2, n (%) | 29 (85.3)  5 (14.7)  0 (0.0) | 19 (54.3)  15 (42.9)  1 (2.9) | **0.008**  **0.015**  0.328 |
| Abbreviations: IQR, interquartile range; PCFS, Post-COVID-19 Functional Scale. ^a^ Difference between proportions in the *Z*-test. ^b^Difference between groups in the Mann-Whitney *U*-test. ^c^During a 3-month follow-up | | | |
